# COVID-19 Pandemic Implications for Corporate Sustainability and Society: A Literature Review

**DOI:** 10.3390/ijerph19031592

**Published:** 2022-01-30

**Authors:** Ruixin Su, Bojan Obrenovic, Jianguo Du, Danijela Godinic, Akmal Khudaykulov

**Affiliations:** 1 School of Management, Jiangsu University, Zhenjiang 212013, China; surx1212@126.com (R.S.); bojan@inovatus-usluge.hr (B.O.); 2 Faculty of Humanities and Social Sciences, University of Zagreb, 10000 Zagreb, Croatia; danijela.godinic5@gmail.com; 3 School of Management, Huazhong University of Science and Technology, Wuhan 430074, China; ahudaykulov@live.com

**Keywords:** corporate sustainability, COVID-19 pandemic, crisis management, leadership, digital transformation

## Abstract

The paper revises the ample empirical and theoretical literature on sustainable organizational growth and strategic leadership relating to the critical aspects of the ongoing pandemic, including poverty, social responsibility, public health, and organizational and managerial innovation. Drawing from available COVID-19, management, and sustainable leadership publications released from 2020 to 2021, this paper considers influential studies exploring core business concepts, principles, philosophies, and activities for accelerating, stimulating, and nurturing social and corporate sustainability. The study analyzed the characteristics and interrelation of 133 articles through bibliometric and literature systemization techniques. We shed light on the significant influence COVID-19 has had on financial, operational, and psychological solvency and organizational health to elucidate expectations and implications for businesses worldwide concerning the long-term financial and functional impact of COVID-19. An overview of the relevant studies on the individual, organizational, and external factors relating to novel disease’s relation to sustainability are provided. We emphasize the need for digital transformation following the COVID-19 upheaval and throughout the upcoming years. Some of the generally employed techniques in response to adversity entail portfolio diversification, service delivery innovation, product redesigning, new market development, partnering with competitors and/or complementary service providers, synergizing with other stakeholders, and open innovation.

## 1. Introduction

The new and unexpected global threat prompted by the SARS-CoV-2 pandemic posed a grave international public health challenge unlike any other in recent history. Declared as an acute and extreme concern by the World Health Organization in January 2020 [1], the disease impeded the fulfillment of UN Sustainable Development Goals (SDGs) with severe implications for social and economic welfare [2,3]. The pandemic originated in November 2019 in Wuhan, China, and rapidly spread all over the world. Without downplaying, causing a threat of cataclysmic proportions, the outburst economic effect is frequently compared to the Great Depression and Second World War [4,5]. In hindsight, the national response to an emergent disaster was to implement impromptu and ad hoc policies that are updated regularly based on the infection’s development and impact on the individual as well as business sustainability [6]. Aside from risks on general health, rapid changes virus provoked in daily functioning and enterprises’ routine operation, as well as organizational citizenship behavior, calls for an elaborated contingency plan for management executives [7,8]. To ensure organizational resilience, business owners are required to consider individual psychosocial effects resulting from fear perception, as well as organizational factors facilitating business transformation and survival [9,10]. Finally, executives will be wise to consider potential external threats impeding their performance and influencing the decision on staying in business vs. closing. All these determinants, collectively, feed into each other and influence sustainability. Therefore, persons in charge are advised to detect risk groups, provide adequate social support, guidance, and training, find the most effective leadership techniques, and intervene when necessary. They are to implement corresponding emergent protective work policies and consider additional funding alternatives to ensure liquidity during the crisis. This literature review aims to provide an overview of sustainability-conducive factors by combining the recent insights from epidemiology, sociology, and human resource management. As it was established many times before that work setting, organization, and job-related behaviors impact employees’ mental health and well-being, it is essential to determine which factors are influenced and further perpetuated by COVID-19 [11,12].

COVID-19 prompted scientists, experts, and health professionals to join efforts in an attempt to find effective ways to mitigate risks, curb the consequences, and balance the trade-offs in a dynamic business environment characterized by sudden changes in market demand unpredictability, financial crashes, and profound adversities [13]. Disastrous effects of COVID-19 encompass severe implications of hardship on public health [14], mortality, poverty and unemployment rates, organizational liquidity [15,16], mental health [17,18], and social and national sustainability [19], as great economic shocks and fiscal contractions followed the implementation of mass lockdowns. The devastating consequences of COVID-19 on the general public are prevalent across all segments of society, and the fallout is especially noticeable amongst vulnerable individuals in low-income countries, although poverty rates are increasingly alarming in all social stratum. Aside from health and economic repercussions, exposure to infection and distress due to fear of unemployment, stigmatization, exclusion, displacement, work-family conflict, and psychological deterioration caused strain on society as a whole, posing a social crisis [20]. Furthermore, lockdown and academic closure posed an additional challenge for the employed parents, as they are expected to take on more responsibilities at home while navigating new roles and tasks in an unfamiliar and disrupted environment [21,22,23,24]. Economic reverberation has a follow-up repercussion for a livelihood by way of limiting available resources, employment opportunities, income, and, consequently, access to healthcare services and essential assets. Protective social policies and measures, as further discussed in this article under the section dealing with external factors influencing sustainability, act as buffers against major shocks by decreasing backlash and temporarily supporting individual and organizational efforts to manage the crisis.

Sustainable development was defined as leveling actions related to the triple bottom line to attain economic, environmental, and social goals and create added value [25,26]. Previous research has characterized sustainability as balancing an integration of economic performance, social inclusion, and environmental welfare for the greater public good [27,28,29]. Sustainable performance was previously hypothesized to be dependent on innovation, strategic orientation, detecting and exploiting the entrepreneurial opportunities, and including cognitive and technological capital for launching novel ventures, products, and operative procedures [30]. The content of organizational sustainability keeps changing and developing to the systematic and strategic management of new knowledge and resources to develop innovative practices for value creation [1]. It hinges on redefining and aligning existing knowledge to foster new ideas and innovative practices to advance sales. The central approach to achieving effective sustainability is through an open innovation, whereby organizations leverage strategic options of introducing new resources or redeploying the existing ones to generate novel products [31,32].

The key concept to consider in the dynamic business environment is agility [33], with its references to leadership, decision making, strategy devising, contingency planning, and responsiveness [34]. Furthermore, business, consumer, and executive behavioral changes entail ethical reflections concerning human resources and public welfare responsibility [35]. To proceed from discourse on the concept of crisis management to its actual applications in a volatile real-world environment for achieving sustainable growth, a unanimous definition stating the basic features concerning its essential dimensions is required. Scholars and policymakers emphasized this need to bridge the gap between social, environmental, and economic bearings. It was previously agreed that sustainable development does not follow segregated initiatives [36,37]. It stems from a synergistic strategy integrating key pillars of sustainability—social, environmental, and economic.

In this article, we explore the relation of COVID-19 to corporate sustainability from the perspectives of both internal organization and external social environment. We try to discuss and find implications for enterprises and society to better cope with crises and achieve sustainable development in the post-epidemic era. There are three issues we mainly focus on. First, how did business reform accordant to novel circumstances, and what was the extent of health threat on business performance? Second, what leadership type proved to be most efficient to ensure enterprise resilience during the emergent situation? Lastly, the study yields a preliminary answer to the question of whether and how does the crisis accelerates digital transformation, e.g., to what extent digital transformation proved to be essential for survival?

The remainder of this study is arranged as follows. Section 2 presents the methods, including classification criteria, search strategies, etc. Section 3 displays the descriptive and co-occurrence bibliometric analysis results. Section 4 records the systematization and grouping of the knowledge generated based on a corporate sustainability model. Finally, Section 5 and Section 6 summarize the direction of future research and practical implications.

## 2. Materials and Methods

This study combined with the bibliometric method and content analysis describes and systematizes the existing literature on the COVID-19 pandemic and enterprise sustainable development following the three-step process [38]. Firstly, it displays the characteristics and interrelationships of related research, then excavates and refines the content and conclusions based on a theoretical model, and finally makes recommendations for future research directions and practices.

### 2.1. Classification Criteria and Search Strategies

**Figure 1:**
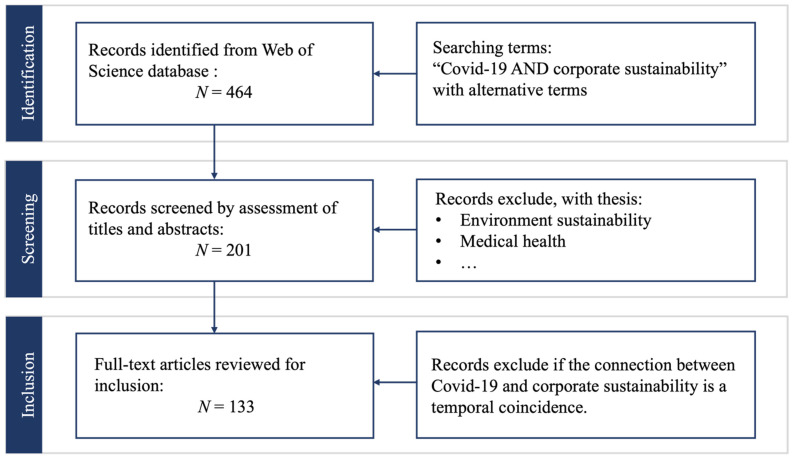
The search strategies and steps.

In an attempt to generate a systematic literature review, we analyzed published work drawing from the concept of “sustainability” during COVID-19, published between January 2020 and July 2021. We identified research objectives and key questions. These were employed as a guidance mechanism in the preliminary selection process and in establishing a proper review protocol by which primary and secondary studies were singled out. Next, we based our study on the notion of sustainability in general and focused on the current context of COVID-19. The search strategies and steps are shown in Figure 1.

The first selection of research was primarily carried out on articles, journals, and conference proceedings retrieved from the Web of Science database. The search terms focused on two central themes of “COVID-19” AND “Corporate sustainability”. In addition, the alternative keywords, key phrases, and their variations were also entered into a digital library and used while searching for articles. Regarding the pandemic, “COVID”, “Pandemic”, “SARS-CoV-2”, and “Coronavirus” were alternative terms [39]. For corporate sustainability, “Enterprise sustainability”, “Organizational sustainability”, “Sustainable business”, and “Sustainable operation” were also included [40]. After the above search, a total of 464 articles were obtained with detailed information, including authors’ names, affiliation, year of publication, journal name, title, keywords, and abstract.

The second step was to exclude out-of-scope themes such as social or national sustainability, environment, and medical health by reviewing both the titles and abstracts. The biggest challenge was that although some articles included “COVID-19” and “sustainability” or their synonyms in the title, keywords, as well as abstracts, they were just a coincidence in time and not deliberately researched on the relationship between the epidemic and enterprise sustainable development.

Therefore, in the final step, the full text of the candidate articles was screened to check for the content relevance to the subject matter. Topics encompassed knowledge domains of crisis management, sustainability, enterprise sustainability, digitalization effects on sustainability, sustainable business practices, COVID-19 business implications, and leadership during COVID-19. Research types included were exploratory, explanatory, empirical, conceptual, and case studies. A total of 133 relevant articles were finally identified and selected for the analysis. 

### 2.2. Method Describing and Systematizing the Literature

The study analyzed the characteristics and interrelation of 133 articles through bibliometric and literature systemization techniques following Cruz-Cardenas et al. [39] and Ranjbari et al. [40]. The bibliometric method displayed the descriptive statistical results of related knowledge and conducted a visual analysis of co-occurrence. Additionally, content analysis was employed to systematize the literature and find a reasonable knowledge organization structure. Content analysis is mainly to help interpret meaning by studying information and features of a text, as well as identifying the frequency of various concepts. This method is one of the most popular methodologies in articles with topics about corporate social responsibility and sustainability [34]. By reviewing the literature on corporate sustainability, this article chose a widely accepted model as the theoretical basis for the following content analysis [34,41].

There are many interpretations for the definition of sustainability, from environmental ecology, economic development, and social tolerance to licensing and other perspectives [42]. Recent studies have shown that organizational sustainability has surpassed the traditional concept “green” and no longer stays at the behavior of, for example, reducing negative environmental impacts, but moves into a more comprehensive and systematic understanding [43,44]. Both researchers and decision-makers realize that it is necessary to transform from traditional business models to strategic, innovative, and sustainable complex systems [41].

**Figure 2:**
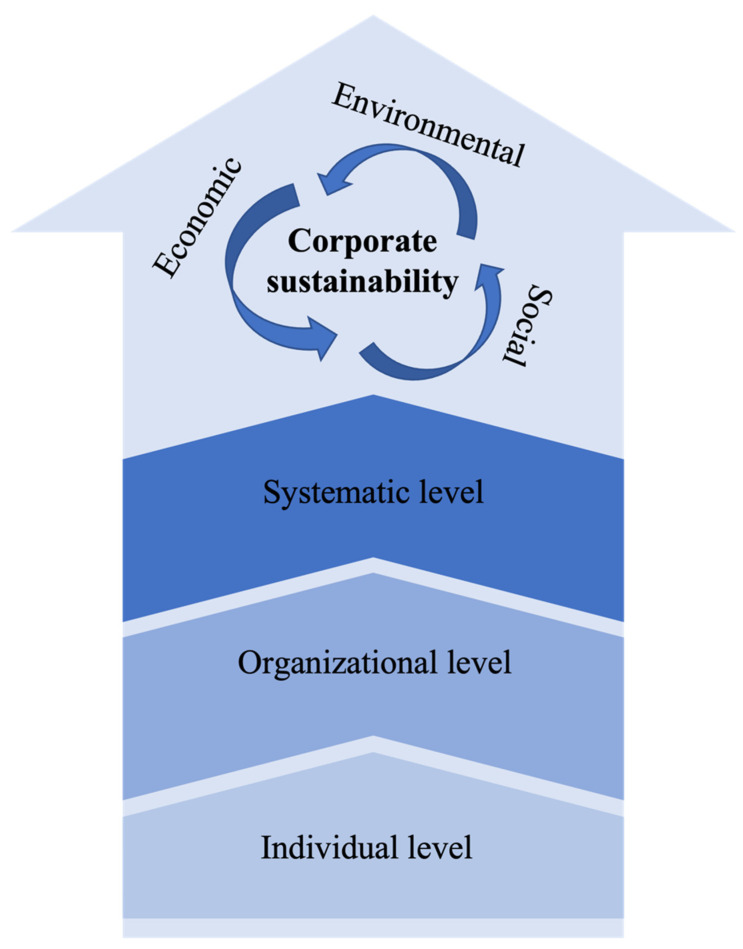
General corporate sustainability model adapted from Ikram et al. [34] and Landrum and Ohsowski [41].

Elkington [45] proposed the concept of the triple bottom line (TBL) to overcome the relatively ambiguous nature of organizational sustainability and simultaneously included the three pillars of sustainability. When applying TBL, companies usually look for areas where these three dimensions overlap and reinforce each other to improve the efficiency of the integrated structure. To further examine organizational efficiency, Ikram et al. [34] and Landrum and Ohsowski [41] combined the individual level, organizational level, and systematic level of the enterprise with TBL and proposed a general model as shown in Figure 2. Our research refers to this model structure for systematic analysis.

## 3. Results

### 3.1. Descriptive Analysis of Relevant Articles

Table 1 lists the journals that publish articles that met our screening criteria. In total, 42 articles were published in the journal Sustainability, accounting for 31.6%, followed by nine articles in the International Journal of Hospitality Management and six articles in the Journal of Applied Psychology, accounting for 6.8% and 4.5%, respectively. Other journals only published articles ranging from one to four articles. Notably, the hospitality industry was the most popular research field, probably because of the infectious characteristics of the COVID-19, which had the most direct and violent impact on the business and operation of hotels. Other industries, studied to a lesser extent, included the medical industry, food service industry, and manufacturing. From the perspective of the size of the enterprise, the attention paid to small and medium enterprises (SMEs) was also very high.

**Figure 3:**
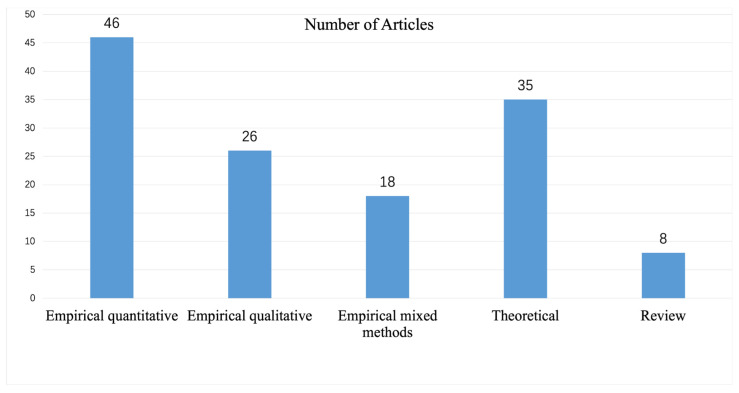
Number of articles according to the methodology.

Figure 3 shows the number of articles per each method. A total of 90 studies employed empirical methodology, accounting for 67.7%, and more than half of them used quantitative methods. Among them, 18 studies employed empirical mixed methods, mainly testing the proposed models based on structural equation modeling. In addition, 35 articles used theoretical analysis, accounting for 26.3%. Overall, the research methods were relatively diverse, mainly quantitative techniques combined with qualitative or theoretical analysis. This may be because, first, quantitative analysis usually takes the form of questionnaires to collect data and then analyzes through mathematical models to obtain fast and conclusive results, while qualitative methods are relatively time-consuming and labor-intensive, and the results may not necessarily meet expectations. Secondly, corporate sustainability is a mature and extensible topic with a large number of theories and evaluation models developed, the depth of which is also suitable for theoretical analysis.

### 3.2. Analysis of the Co-Occurrence and Cluster Identification

**Figure 4:**
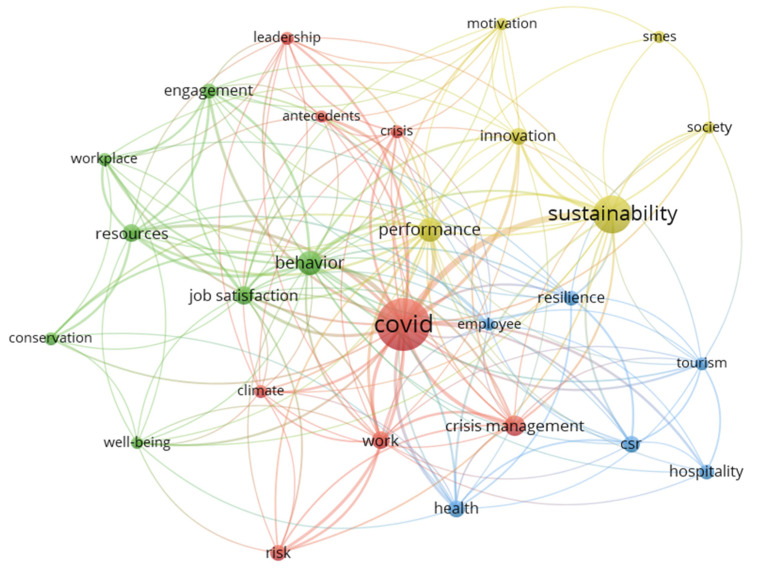
Co-occurrence network of articles based on author keywords.

**Figure 5:**
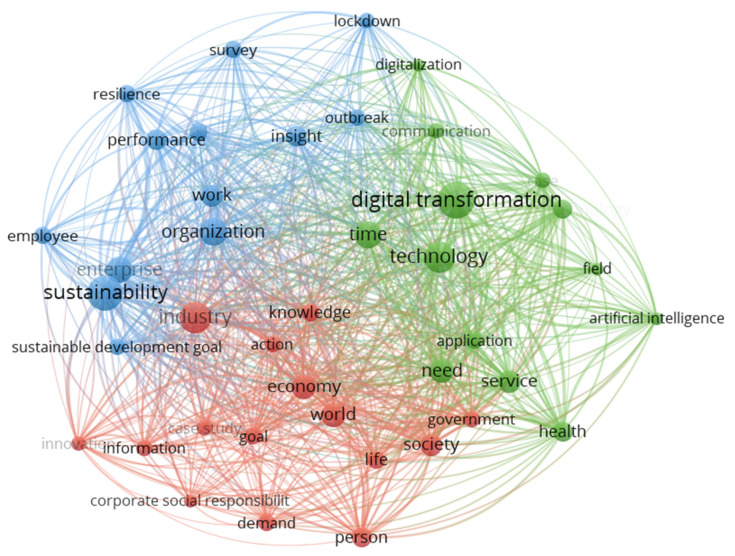
Co-occurrence network of articles based on titles and abstracts.

The study used co-occurrence analysis to identify key research topics in COVID-19 and corporate sustainability-related research. This analysis was performed based on keywords and based on titles and abstracts for more reliable results by VOSviewer 1.6.17 [46]. VOSviewer is a common tool for analyzing bibliometric networks. It can build maps of publications, titles, authors, keywords, or abstracts based on a co-citation network. VOSviewer uses different colors to represent each cluster of keywords or concepts, with nodes representing keywords or concepts and their sizes corresponding to the frequency. Compared with other literature analysis tools, although it cannot view node information or perform time slicing, it is easier to operate and can produce clear and vibrant images, which can better display the results of clustering. In our analysis, generic terms such as “research”, “author”, and “methodology” were eliminated during data cleaning. Similar terms are grouped into one, such as “COVID-19”, “Coronavirus”, and “Pandemic” [46]. Phrase abbreviations were also noted and replaced, for example, “small-medium enterprises” and “SMEs”. In addition, VOSviewer recommends that the minimum number of occurrences for a term is five, yet due to the relatively limited number of articles used in the analysis, we followed Cruz-Cardenas et al. [39] and changed the number to three for a better result. Figure 4 and Figure 5 are the obtained clusters generated by core data.

There is no doubt that COVID-19 and sustainability are the most frequent keywords. Cluster 1 (red) has COVID-19 as its prominent node and gathers other keywords like work, crisis management, antecedents, and risk, which can be marked by epidemic hotspots. The central node of Cluster 2 (yellow) is sustainability and groups together with performance, innovation, and motivation. This cluster is related to corporate performance and innovation during the COVID-19 pandemic. Cluster 3 (green) has behavior as its central node with job satisfaction, engagement, workplace, and other relative phrases, which can be strongly labeled as employee behavior. Although cluster 4 (blue) has no central node, terms like hospitality, tourism, and resilience imply that the service industry has received more attention, which is consistent with the results of the previous descriptive analysis.

In addition, to explore in-depth the research topic and scope of the selected literature, another co-occurrence analysis was performed based on titles and abstracts. Similar to the previous screening process, after reasonable cleaning of the data and substitution of synonyms, the results are shown in Figure 5. There are three clusters. The first cluster (blue) revolves around sustainability with other keywords such as organization, performance, work, employee, and resilience. These are all key elements for organizations to sustain sustainable development during the COVID-19 pandemic. The second cluster (green) is centered on digital transformation, accompanied by technology, communication, digitalization, service, time, etc. It indicates that the epidemic has encouraged the development of digital transformation and technology innovation. The third cluster is red and has no core points, but keywords such as economy, world, industry, society, life, and government show the macro external economic, social, political-legal environment influences.

Comparing the results of the two co-occurrence analyses, it is found that the conclusions are similar and coincide with the previous descriptive analysis, which once again proves the correctness and credibility. Therefore, the impact of the COVID-19 pandemic on corporate sustainability can be developed from three aspects: first, from the individual level, the effect on employee health, workload, environment, and job satisfaction; second, the severe shocks on organizational crisis management and business innovation, especially in the service industry; finally, from a socio-economic perspective, the external environment has also fanned the huge impact of this epidemic.

## 4. Systematization of the Relevant Literature

This section presents the content analysis and systematization of the 133 selected articles based on the structure of the corporate sustainability model in Figure 2. Implications of the pandemic on enterprise sustainability attracted the attention of many scholars. The research perspectives range from internal organization to external environment, from human resource to operation management, from a single business to supply chains, and from direct impacts to indirect ones.

### 4.1. Individual Level

Due to its long-lasting consequences for social and economic welfare, the new COVID-19 pandemic has posed a global challenge for public health and business sustainability. Due to the high contamination risk, many entrepreneurs and managers have introduced telecommuting [47]. Notwithstanding the benefits for physical health maintenance in terms of prevention via safety distancing, social isolation, and remote working, new challenges are prompted to employees’ mental health [20]. Concerns such as social exclusion, isolation, solitude, work overload, uncertainty, and disturbed work–life balance emerge [48]. Not only have emergent work policies affected employee psychological health, but resulting deterioration in psychological well-being and mood swings are found to mediate the work performance, and consequentially, business sustainability [49,50,51]. Stress, burnout, and psychological side-effects can be felt and measured both at the individual and organizational level. Considering they are prevalently measured through employees’ self-rated reports, in this part, we provide an individual-level overview of coronavirus ramifications for mental health that ultimately influence organizational sustainability.

As the pressure on workers increases, COVID-19 has produced a paradoxical effect with an equally detrimental impact on organizational sustainability [52]. First, the increased paranoia and fear of infection causes absenteeism from work. Second, the fear of downsizing and unemployment causes presenteeism, wherein sick individuals do not take a leave of absence despite the infection [53]. The relationship between COVID-19 fear and the latter is mediated by leadership variables, as employees follow their management’s example [54]. As it was established many times before that work setting, organization, and job-related behaviors impact employees’ mental health and well-being, it is essential to determine which underlying factors are influenced and further perpetuated by COVID-19. It is crucial to identify the alleged stressors on employees’ psychological welfare to alleviate the adverse effects and ensure wellness. Furthermore, it is also imperative to decide which mental health outcomes feed back into organizational culture, infringing work–life balance and translating to negative organizational citizenship behaviors.

The factors affecting the mental health of employees include work overload and job stress [20,55,56,57]. For example, Lai et al. [58] conducted a cross-sectional study to explore the mental health outcomes prompted by COVID-19. The surveyed population consisted of health care workers from 34 hospitals in China. Study results showed that most respondents experienced depressive episodes, anxiety, insomnia, and increased distress due to extended workload, increased media coverage, lack of drugs, and lack of social support. Over 70% of workers reported feeling distressed, and authors speculate that anxiety stems from lack of control, concerns about the magnitude of infection, high morbidity rates, and increased risk perception. Similarly, the triggers of increased psychological pressure promoted by [59,60] were loneliness and helplessness, dysphoric emotion, stress, and mental fatigue from work overload and burnout.

The increase in workload brought by the remote working model has also broken the balance between work and family [21,22,23,24]. The guidelines on managing work-related psychosocial risks during COVID-19 published by International Labor Organization (ILO) lists that fear and the way it relates to employees’ perception concerning the likelihood of infection, unemployment, layoffs, and poverty is identified as the main driver of psychological distress. Furthermore, working from home setting undermines the delicate work–life balance, as family interference, reinforced more so by educational institutions’ closures, can create pressure for parents. Furthermore, some workers are ipso facto additionally exposed to the hazards of domestic violence. Stress factors, in turn, lead to frustration and anger and act as violence, discrimination, and harassment antecedents.

In addition, external environmental influences such as media exposure and social panic also put a lot of pressure on employees’ psychology [61]. Main stressors are divided into the following categories: perception of safety, threat and risk of contagion, infobesity versus the unknown, quarantine and confinement, stigma and social exclusion, as well as financial loss and job insecurity. Some researchers ascertained that organizational, institutional, and individual factors mediate the relationship between COVID-19 and mental health, institutional being diverse governmental programs and financial aid packages that reduce fear perception [52,60,62].

In this severe situation, the negative mental health of employees undoubtedly affects the quality of tasks, thereby affecting corporate performance and business sustainability [14,63,64,65]. Sasaki et al. [14] concluded that the fear of COVID-19 and psychological distress could affect work satisfaction and turnover intention. Consequently, clear response workplace measures are beneficial to the maintenance of occupational and mental health. In addition, stress levels were found to be lower when employees were trained on managing the risk of COVID-19, and this, in turn, resulted in enhanced job performance and job satisfaction and decreased turnover intention. Sufficient training and knowledge sharing can increase employees’ confidence in their knowledge and capability to execute preventive actions successfully [66,67,68].

In summary, this part provides an overview of sustainability-conducive factors from the perspective of employees by combining the recent insights from epidemiology, sociology, and human resource management. A large number of articles use empirical methods [69,70,71], especially structural equation models [72,73,74], to explore the factors that led to the mental health problems of employees during the epidemic: (1) the biggest change for employees is working remotely. This work model brings problems such as low communication and collaboration efficiency and uneven distribution of tasks, which increase the workload and anxiety of employees; (2) the broken balance of work and family; (3) external media exposure, lack of drugs, insufficient social assistance, and other negative information have caused fear and work insecurity; (4) social interaction is restricted, followed by blocked communication between relatives and friends; (5) competition in the labor market is intensified with the emergence of inequality. All these factors make employees fall into negative emotions such as anxiety, fear, loneliness, worry, and collapse. This, in turn, affects job performance and employee satisfaction and then poses dangers to sustainable human resource management (HRM).

### 4.2. Organizational and Systematic Levels

#### 4.2.1. Leadership

Sustainable leadership is one of the key variables in the turbulent business environment that helps address market behaviors and advocate proactivity. Moreover, leaders’ qualities add up to encouraging employees to perceive changes in the external environment and sudden rises and fall in demand spikes as learning opportunities rather than insurmountable challenges [75]. Management is, therefore, tasked with reassuring employees and boosting their self-efficacy beliefs and, consequently, their working capabilities [76,77,78,79].

From the perspective of leadership types, personal characteristics, and behaviors, considerable literature reaches a similar conclusion that full empowerment, task orientation, and entrepreneurial leadership are essential in such volatile environments [19,77,79,80,81,82,83]. For instance, Obrenovic et al. [19] emphasized the importance of delegating authority and endowing followers with a certain amount of autonomy in decision making, as this helps build confidence and strengthens the organizational commitment and accountability for one’s actions. Leaders should be risk-prone yet experienced and confident. Coincidentally, Bucher et al. [81] maintained that leaders should be supportive, provide guidance, act transparently and fairly, and value followers’ input. Furthermore, leaders must share intelligence, skills, and knowledge with followers and provide training to facilitate decision making and optimal employee’ contribution to sustainable organizational performance. Leaders should possess both entrepreneurial orientations focused on fostering innovative work behavior and empowering qualities. In addition, engaging in task-oriented behavior with clear guidance improves teamwork in virtual settings while empowering followers with autonomy and support allows for better adaption to novel crisis yielded circumstances. Digitally mature firms are more likely to sustain performance during the crisis [84].

Entrepreneurial leadership is defined as a type of leadership emerging from the crossroads between entrepreneurship and management that focuses on harboring heterogenous talents and engaging in creative and innovative collaboration within the organization as a response strategy to unstable and tumultuous market conditions [28,85]. Nor-Aishah et al. [28] incorporated innovation as a defining feature of practicing entrepreneurial leadership, as it pushed organizations towards risk taking, improvising, creating, and innovating. They conducted a cross-sectional study on 146 respondents from Malaysia to explore the relationship between entrepreneurial leadership and economically sustainable performance, environmentally sustainable performance, and social sustainability performance. The study findings confirm the impact of entrepreneurial leadership on environmentally sustainable performance and sustainable social performance. The authors concluded that entrepreneurial leadership is essential for achieving sustainable performance during catastrophes. Stated results are consistent with the studies by [86,87,88].

Studies have also shown that antecedents of organizational change commitment through digital transformation include trusting leadership–follower exchange, high autonomy, and improved self-efficacy [84]. Digital transformation requires, besides a necessary infrastructure and connectivity, a high level of technological proficiency, and all employees need to precisely understand their novel roles and tasks to ensure there are no hindrances and render smooth transition into a new environment. According to the empirical analysis of Jung et al. [89], empowering leadership was positively associated with commitment to organizational change, while risk-taking leaders’ behavior positively mediated the relationship between empowering leadership and employee commitment to organizational change. Furthermore, especially for SMEs, the primary step to maintain sustainability is to employ a comprehensive digitalization strategy, whereby it is necessary to foster a clear understanding of digital technology, appoint appropriate leaders, launch a superior digital business center, define a digital strategy, gather and develop new technology-related knowledge, and create new digital capacities. In this, organizations should combine human resources with technology [84,90].

In short, the above review of the literature suggests that leaders and business owners will, in large, be successful in ensuring organizational sustainability if they introduce (1) task orientation, (2) full empowerment, (3) entrepreneurial leadership, (4) innovation, and (5) technological improvements.

#### 4.2.2. Management and Operations

During uncertainty, in addition to strengthening human resource management, executives and business owners must also align coexistent business management strategies and operations to uprising marketing needs. Research on most successful generic strategies employed during disasters boils down to a variation of a few variables, namely, crisis management, product diversification, financial flexibility, supply chain, technological innovation, and digital transformation [91,92,93,94]. Business as usual shifts to an emergency model. Multiple techniques are deployed simultaneously to meet the changing needs, either by adding extensions to an existing product line through product diversification, endorsing new methods to utilize existing capacities, or innovative production [33,95].

First of all, some articles focused on the overall management and control of enterprises [96,97,98,99]; for example, Hao et al. [97] developed novel disaster management during the pandemic framework. The initial motivation behind their efforts was to develop an effective managerial framework consisting of several measures and procedures tailored to each of the six disaster management phases intended for the hospitality industry. Yet, their strategies can have a far more extensive application. They found that interventions in leadership and communication, HR, service provision, CSR, finance, and disaster management standard operating procedure, when combined, lead to a uniform and effective strategic game plan. In addition, combining technological innovation in the framework can help organizations respond more quickly. Lee and Trimi [99] conducted a study on the relationship between advanced technologies and innovation during disasters to determine the effect of combining technological potentials with creativity on value creation. They emphasized the relevance of sustainable innovation and its priority in achieving organizational survival, as the evidence they generated showed that convergence innovation would lead to innovation management strategies.

Some literature focuses on financial flexibility [16,100,101]; for example, Teng et al. [16] considered financial agility to be the pillar of resilience and organizational sustainability amidst the current pandemic. Liquidity and flexibility allowed enterprises to respond to unexpected shocks in cash flows effectively. Sustainability was achieved through financial flexibility in the asset-heavy manufacturing industry, while the asset-light industry’s effect was not significant. Zimon and Dankiewicz [100] proposed that enterprises could achieve financial liquidity by changing trade credit management strategies from moderately conservative to highly conservative.

Marketing channel plays a vital role in crisis management and business continuity during and post-crisis strategies, which also attract the attention of many scholars [102,103,104]. Engaging in multiple synchronous strategies helps sustain operation and enables enterprises to prosper post-crisis. By reconceptualizing and redeploying the Omni-marketing channel approach, a new operation strategy may be devised wherein multiple distributors, suppliers, and sales points contribute to revenue generation. Several processes should be undertaken simultaneously for the full impact, namely, shortening of the supply chains, creating novel products to suit the emergent market needs, benefiting from digital marketing, initiating collect on delivery transactions, and enabling of e-wallet.

When referring to supply chain, Karmaker et al. [105] examined the sustainability of supply chains during versatile COVID-19-induced conditions. They delved into the antecedents of supply chain resilience during disruptions in Bangladesh. Their study indicated governmental financial aid relief and support from supply chain partners were necessary to resist the economic shocks. Sustainability also drew from health protocols standardization and operational and knowledge management processes automatization. In the same line, Sarkis [106] inquired into supply chain sustainability by examining successful case studies and publications, including the information retrieved from virtual open forums and interviews. Environmental sustainability practices may have a critical role to play in improving organizational competitiveness throughout the novel ecosystem, while technological innovations have an enabling role in crisis management. In other words, big data, decision-making tools, and emergent collaborative blockchain technologies spur supply chain sustainability [107]. Technological innovations are assumed to facilitate and foster the agility of the supply chain [108,109,110].

Since small and medium-sized enterprises are more vulnerable and lack liquidity, how they survive the blow of this pandemic has been more worthy of attention [13,15]. Additionally, from an industry perspective, the service industry represented by hospitality has suffered the most direct impact from the epidemic [111,112,113]. Therefore, their sustainable developments have become a research hotspot. Bartik et al. [15] examined the impact of COVID-19 on small businesses operations and performance weeks after the onset of coronavirus. They surveyed more than 5800 SMEs during 2020, checking for a few variables—business operations, financial implications, and beliefs concerning the long-term impact and future projections of pandemic duration. They found the risk of closure negatively associated with the crises’ expected duration and major discrepancies among business owners’ beliefs concerning the disruptions. Furthermore, SMEs were found to be financially fragile and lacking the liquidity to sustain their operations without receiving government financial aid.

Therefore, current scholars not only propose corporate crisis management frameworks based on information system technology to help companies achieve rapid response and handling of crises, as well as maintain and promote the realization of sustainable goals, but also carry out targeted in-depth research from different perspectives of HRM, financial flexibility, marketing strategy, supply chain, technological innovation, and others.

#### 4.2.3. Digital Transformation

Many scholars and executive managers accentuate digital transformation as one of the first protective measures for ensuring sustainability during all major disasters that include cutting down communication, supply, and delivery and restricting physical functioning [21,114,115,116,117,118]. Possessing sufficient technological solutions, infrastructure, and know-how is a key remedy against adversity, as it allows re-establishing normal operation and communication with all relevant stakeholders, sustains business activity during physical restrictions and lockdowns, facilitates effective and rapid response to emerging challenges, and even provides the opportunity for redesigning of antiquated processes and business models to expand company portfolio and extend product offering. Digital transformation leads to the implementation of advanced automated solutions and service innovation, enhances the management and sharing of business intelligence, and helps organizations enter a new digital market and unexplored customer segments [119]. A new crisis brings on a race with time—equivalent digital products and services are to be created and sold to customers to sustain basic enterprise liquidity. In such a context, most studies focus on exploring the necessity of digital transformation, either as an isolated strategic move or in combination with other key tactics [120,121,122]. Digital transformation is usually considered as one of the few fundamental pillars of a sustainable and digitally literate society, and many countries readily invest large amounts of assets in technological progress [123,124]. Therefore, one of this study’s key objectives is to examine whether and to what extent has the COVID-19 pandemic accelerated the digital transformation as well as its role in dealing with a crisis.

Researchers pay more attention to the digital transformation of SMEs, not only promoting the building of future digital business economy but also suggesting different digital transformation paths [114,125,126]. The best strategy to attain resilience is to transit to e-commerce, online promotion, online sales, and logistics. SMEs can implement cloud-based technology for monitoring operations, accessing financial reports, and tracking inventory and sales. Furthermore, technology facilitates the workflow during COVID-19 and provides opportunities for innovation. Connectivity, functional use of the internet in design, manufacture, promotion and sales, security, simulation, and blockchain are identified as main digital transformation constituents [127]. Researchers conclude that SMEs can attain sustainability by transferring to an online environment where they can build digital business models and use IT for evaluation and digital value network designs. About the transformation paths, digitally mature SMEs respond by accelerating the transition towards digitalization; financially challenged SMEs with low digital maturity digitalize sales solely, while a high level of social capital supports SMEs with limited digital literacy, and they often team up with partners in possession of excellent digital capabilities.

By using digital resources, the economy has proceeded from geographically constrained to the actual global economy, whereby organizations combine versatile web-based technologies such as apps, platforms, and marketplaces to increase their sales and attract more customers. Digital transformation has a varied effect on diverse industries, yet its impact is undeniable. The industry’s very concept is modified and has moved from the idea of industries as stable competition domains to dynamic competition due to digitalization [128]. Technology innovation diminishes the barriers among industries, and organizations can now participate in more than one segment due to the power of the Internet, while tech businesses are extending their focus on additional products. Although e-learning, teleworking, and e-commerce have existed long before the outburst of a novel virus, the pandemic has now accelerated the transformation of traditional organizations at all levels as it was the only available way for maintaining regular operations [129]. A large number of studies indicate that COVID-19 has profoundly influenced the digitalization process and pushed forward the acceptance and implementation of technology as an adequate solution to avert disruptions and, consequently, economic collapse. They also consider how online learning, digital strategy, artificial intelligence, instant messaging, social interaction, big data, and privacy affect the company’s reconceptualization in the new digital environment [130]. Furthermore, some authors believe COVID-19 to be the catalyst for creating digital startups and new digital value propositions. Digital transformation comes with many benefits for sustaining enterprises, such as smart tools and knowledge management systems, automated order processing, advanced CRM tools, ERP, cloud solutions and IoT, smart manufacturing, and blockchain [128,131].

### 4.3. Macro-Environmental Factors

For COVID-19 and corporate sustainability, the following five macro forces should not be underestimated: the epidemic and the technological, social, economic, and political-legal environments. Great importance is attached to the technical environment. As summarized above, COVID-19 is closely related to digital transformation based on technological innovation. It can even be said that the epidemic has promoted technological progress and accelerated innovation and transformation at all levels of the enterprise.

In addition, economic and social inequality has been increased during this epidemic [132,133]. Susceptiveness to infection is greater in low-income countries (LIC) due to existing poverty, poor nutrition, and inadequate health care [134,135]. Furthermore, unlike the rich economies, low-income countries with remote communities experience lockdowns more profoundly, as they face a threat to food security. Adhering to social distancing measures is next to impossible since most people live in crowded houses and cannot afford additional accommodation. All these external circumstances anticipate and affirm pre-existing economic and social inequality. Fiscal solutions to issues arising from temporary termination of work attendance are only provisional and short-term and not enough to sustain economic and organizational functioning due to the discriminatory social policies [136,137]. According to UN and IMF estimations, the pandemic is expected to accelerate inequality, pushing 8% population into poverty. Some countries have arbitrarily managed the allocation of medical workers and PPE equipment based on contingent risk assessments, thus violating the International Health Regulation and 2005 Pact on collective action. This series of economic and social inequalities also exacerbate existing inequalities in the labor market and impact organizational human capital [13,138], especially concerning the already uncertain status of women, immigrants, and young employees. Inequalities are characterized as providing resources and growth development for certain employees and enterprises, respectively, while simultaneously intensifying salaries and work-related benefits for others. Deriving from the previous studies on the subject matter, authors conclude there is a bidirectional relation between organizational and societal inequalities, which are expected to grow in the aftermath of COVID-19 rapidly. Such an unfavorable trajectory will spill over to the workplace, causing burnout, absenteeism, neglecting the norms, discrimination, attendance, and turnover.

**Figure 6:**
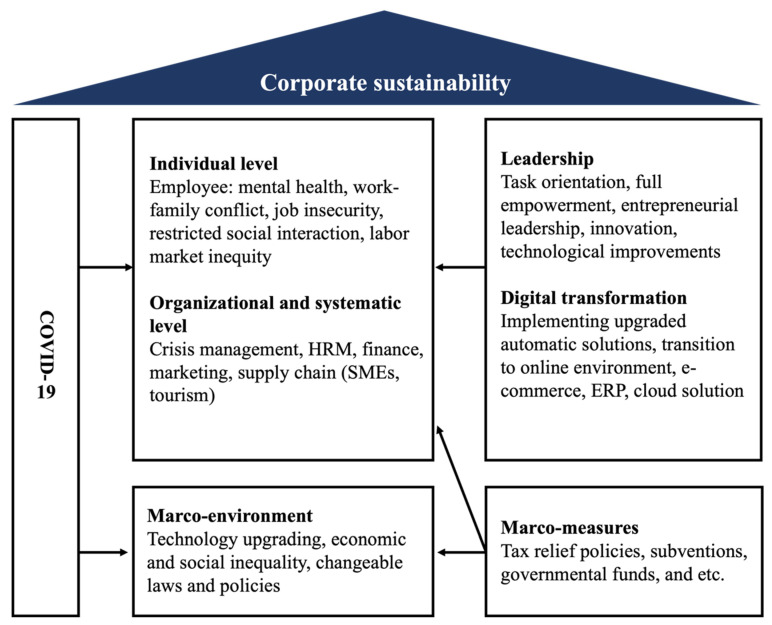
Key factors conducive to corporate sustainability.

From the perspective of the political–legal environment, external factors relating to the COVID-19 pandemic encompass the comprehensive implementation of international measures undertaken to support overall national sustainability, ensure employment, and provide compensation for workers and entrepreneurs alike. These include various tax relief policies, subventions, governmental funds, social security contributions, and reimbursements [139]. The emergent policies may be categorized as expenditure measures, tax measures, sectorial and regional measures, measures other than fiscal, and all other measures. Policies introduced, budgetary amount, and budgetary impact vary among countries. As the ongoing emergent condition is still evolving, listed policies are subject to being updated, amended, or terminated. A decrease in available human resources and labor shortages, gathered with inequality, signaled the need to rely heavily on public and private debts [13]. Therefore, the government’s fiscal aid has become the reliance of individuals and organizations. For individuals, temporary unemployment due to the outbreak is considered temporary unemployment in the case of force majeure. It is also applicable to organizations facing difficulties due to accelerated infection, especially those that experienced partial or full activity suspension due to lack of liquidity. In this situation, temporary unemployment benefits from a government agency, as the EU commission suggested, including the allowance for young unemployed, are extended over a few months during the lockdown, and reimbursement for ensuring continuous employment is activated, where the government assumes the cost of employee salaries [140,141]. On the organizational level, most of the EU member states have adopted updated tax measures whereby tax deferrals are facilitated due to financial difficulties for organizations and self-employed populations, tax is postponed, and entrepreneurs are exempted from paying social security for the time being [142]. Therefore, government financial aid is essential for combating the financial distress prompted by immediate economic shocks and maintaining sustainable business development during the COVID-19 pandemic [105]. Especially among SMEs, considering the relative lack of liquidity and, consequently, general fiscal fragility, many enterprises were eligible for additional funding and loans but had difficulty substantiating the qualification [143,144]. Funding in large influences their future actions regarding potential layoffs and downsizing and is bound to overweight when determining whether to continue with operating or shut down businesses [15]. However, the COVID-19 crisis is dynamic. Since many governments have now stopped lockdown measures and large-scale vaccination has been carried out steadily, the epidemic continues to affect the stability of individuals, organizations, and the entire society. The key factors conducive to corporate sustainability are presented in Figure 6.

## 5. Discussion and Implications

Most of the studies conducted on the business implications of COVID-19 focused on organizational factors such as leadership, management, and digital transformation. In comparison, there is significantly less empirical work on individual and external factors affecting enterprise sustainability. Individual factors influencing one’s psyche and general effectiveness, including the effect of uncertainty on anxiety, fear, psychological functionality, and well-being, are inspected through the prism of the general population’s psychological and health implications of coronavirus. These are yet to be linked to workplace performance and organizational resilience. Some of the studies have focused on the effectiveness of telecommuting and remote work on organizational performance. Although the concept is not new, extraordinary circumstances and additional plausible impedances perpetuated by COVID-19 may have affected the causal relationship, rendering previously generated evidence outdated or obsolete. Therefore, further research on remote work in the context of COVID-19 should be carried out.

In addition, out of all the studies examined in this review, the majority have focused on the possibility and relevance of digital transformation for businesses during COVID-19, while the amount of research on distinct leadership styles is significantly lower. Furthermore, in comparison to an examination of the effects of digitalization, leadership analysis was mostly conceptual, lacking empirical proof for hypothesized models. Articles related to successful business practices pose a myriad of management strategies, and there is a clear lack of consensus among the authors on the drivers of sustainable operation.

Future studies can also pay more attention to the application of new theories and the identification of other factors that can be included in the theoretical model. For instance, a new perspective using leadership and behavioral theories could examine types of leadership, individual characteristics, and behaviors of leaders and workforce during the COVID-19 pandemic. Furthermore, a deeper investigation into particular economic sectors warrants more research. For large and financially viable enterprises, the COVID-19 crisis posed an opportunity for entering new avenues and market segments, whilst SMEs lacking resources for such maneuver required cost-effective and quick-fix plaster solutions.

Furthermore, while the majority of the studies on sustainability and liquidity during COVID-19 state that government relief aid for sustaining jobs is a significant contributor to ensuring viability, this is general information rather than a well-examined causal factor. Actual proof of the impact of financial aid and sustainability should be provided, considering many organizations have reduced the number of employees or unnecessary processes to sustain their operations despite the government’s funds, either because the loans were intended for short-term, or the amount was not sufficient. More research should be conducted to link the public monetary relief to variables such as technological improvements, the opportunity for innovation, and sustaining jobs. Such studies should be backed by substantial empirical evidence. Future research could investigate political and environmental variables impacting organizational sustainability in the context of COVID-19, especially regarding specific government restrictions.

## 6. Conclusions

As the most severe crisis for the global society in 2020, COVID-19 has had a huge impact on a wide range of businesses and industries. This has attracted the attention of scholars, but this short-term agglomeration effect has also led to fragmentation of the literature [40]. Therefore, this paper reorganized and reviewed the relevant literature on the epidemic and corporate sustainable development and explored both the challenges and opportunities from the internal organization and external economic environment. We found that a series of changes in work styles during COVID-19 affected the mental health of corporate employees, bringing anxiety, depression, job insecurity, and other negative emotions. In addition, working from home not only deepened work and family conflicts but also reduced communication and work efficiency and then affected corporate performance. The epidemic also had influences on the corporate financial liquidity, market channel expansion, supply chain stability, and operation efficiency, especially in SMEs and the tourism industry. In this context, the role of leadership is crucial. We found that entrepreneurial leaders can adapt more quickly to changing environments and guide companies to maintain sustainable development. Other important characteristics and behaviors of leaders included task orientation, full empowerment, and innovation. At the same time, digital transformation should be grasped to realize the innovation and upgrading of the internal systems and to improve agility and coping ability. The influential external environmental conditions encompass the upgrading of technology, economic and social inequality, and changeable laws and policies. Therefore, if companies want to maintain sustainable development in the post-COVID-19 era where challenges and opportunities coexist, they must fully integrate internal and external resources and rely on digital transformation to achieve survival, development, and upgrade.

Therefore, although the COVID-19 pandemic has brought a huge shock to organizational sustainability, it is worthwhile to notice that it may offer businesses a great opportunity to transfer to genuine corporate social responsibility and ethical decision making [104]. The key management, institutional, and governance factors leading to more responsible business practices include donating financial aid, investing in CSR, adhering to consumer ethics, and engaging in innovative thinking. All these actions may lead to an unprecedented future of corporate sustainability combining environmental, social, economic values [115].

While much of the upcoming changes are problematic to foresee, it is plausible that these developments will profoundly affect fundamental business foundations, philosophies, and processes [145]. In the aftermath of the pandemic, a new ecosystem will likely emerge where policymakers, public authorities, consumers, suppliers, manufacturers, and providers, including previously competing entities, all collaborate and extend their services for the greater good in coherence with the principles of corporate social responsibility [146,147]. All stakeholders are expected to engage in unprecedented exchange, business and otherwise, of intelligence, information, advice, expertise, knowledge, services, and even investments to ensure national growth, keeping in mind that ignorance is the cause of fear. More precisely, we are entering the era of business strategizing based on principles of what Seetharaman [148] refers to as “temporal adhocracies”. 

In the research paper paper, only content analysis of 133 research papers has been conducted, resulting in a qualitative understanding of the studies. The research papers used are summarized in Table 2. 

Future studies could also use a meta-analysis approach to studying the COVID-19 pandemic in regard to corporate sustainability. Another limitation is that we only use VOSviewer software for co-keywords network analysis to get the topic clusters and research trends. However, the analysis of the direction and strength of the relationship between COVID-19 and corporate sustainability is insufficient. Future research may utilize richer software and methods for in-depth analysis, such as CiteSpace, the Loet Leydesdorff disciplinary overlay toolkit, and CitNetExplorer, or consider whether there exists the statistical significance between these two subjects. Furthermore, future studies can focus on testing the impact of various strategies such as product diversification, sustaining radical innovation in service delivery, and open collaboration with market leaders on sustainable development during the COVID-19 pandemic. During the COVID-19 pandemic, many businesses have reached for rather creative growth strategies, focusing either on selling current products and services to new market segments, selling new products to the existing consumer base, or combining the two and detecting and mapping diversification opportunities. Such business should be investigated in the future to determine to gather empirical evidence on the best practices during the COVID-19 crisis. Small-scale temporary changes or more fundamental, permanent business re-organizations should be explored.

To provide a revealing and informative basis for economists, psychologists, policymakers, and marketers to build upon when devising and testing for efficiency of ad hoc managerial strategies, we have recapitulated some of the latest conceptual and empirical evidence on the success of distinct business techniques.

## Figures and Tables

**Table 1:** Journals in which reviews articles were published.

Journals	Num.
Sustainability	42
International Journal of Hospitality Management	9
Journal of Applied Psychology	6
International Journal of Environmental Research and Public Health	4
Frontiers in Psychology	4
Journal of Cleaner Production	3
International Journal of Information Management	3
Science of the Total Environment	2
Population Health Management	2
Journal of Service Management	2
Journal of Business Research	2
Jama Network Open	2
International Journal of Operations & Production Management	2
International jJournal of Contemporary Hospitality Management	2
Environment Development and Sustainability	2
British Food Journal	2
Other journals	44
Total	133

**Table 2:** The literature used for analysis.

Topics	Sub-Topics	No.	References
Individual level	Employee	37	[9,10,14,17,18,20,22,23,24,47,48,49,50,51,52,53,54,55,56,57,58,59,60,61,62,63,64,65,66,67,68,69,70,71,72,73,74]
Organizational and systematic level	Leadership	18	[19,28,75,76,77,78,79,80,81,82,83,84,85,86,87,88,89,90]
	Management and operations	33	[7,8,11,12,15,16,33,34,35,42,91,92,93,94,95,96,97,98,99,100,101,102,103,104,105,106,107,108,109,110,111,112,113]
	Digital transformation	19	[21,114,115,116,117,118,119,120,121,122,123,124,125,126,127,128,129,130,131]
Macro-environmental level		26	[1,2,3,4,5,6,13,39,40,132,133,134,135,136,137,138,139,140,141,142,143,144,145,146,147,148]

## Data Availability

Data sharing not applicable.
